# Identification of marine natural product Pretrichodermamide B as a STAT3 inhibitor for efficient anticancer therapy

**DOI:** 10.1007/s42995-022-00162-x

**Published:** 2023-02-06

**Authors:** Rui Li, Yue Zhou, Xinxin Zhang, Lujia Yang, Jieyu Liu, Samantha M. Wightman, Ling Lv, Zhiqing Liu, Chang-Yun Wang, Chenyang Zhao

**Affiliations:** 1 School of Medicine and Pharmacy, Institute of Evolution & Marine Biodiversity, College of Food Science and Engineering, Ocean University of China, Qingdao, 266003 China; 2 Laboratory for Marine Drugs and Bioproducts, Qingdao National Laboratory for Marine Science and Technology, Qingdao, 266237 China; 3 Department of Cancer Biology, Lerner Research Institute, Cleveland Clinic, Cleveland, OH 44195 USA

**Keywords:** Pretrichodermamide B, Signal transducer and activator of transcription 3 (STAT3), Marine natural products, Target protein, In vivo anti-cancer efficacy

## Abstract

The Janus kinase (JAK)/signal transducer and activator of transcription 3 (STAT3) regulates the expression of various critical mediators of cancer and is considered as one of the central communication nodes in cell growth and survival. Marine natural products (MNP) represent great resources for discovery of bioactive lead compounds, especially anti-cancer agents. Through the medium-throughput screening of our in-house MNP library, Pretrichodermamide B, an epidithiodiketopiperazine, was identified as a JAK/STAT3 signaling inhibitor. Further studies identified that Pretrichodermamide B directly binds to STAT3, preventing phosphorylation and thus inhibiting JAK/STAT3 signaling. Moreover, it suppressed cancer cell growth, in vitro, at low micromolar concentrations and demonstrated efficacy in vivo by decreasing tumor growth in a xenograft mouse model. In addition, it was shown that Pretrichodermamide B was able to induce cell cycle arrest and promote cell apoptosis. This study demonstrated that Pretrichodermamide B is a novel STAT3 inhibitor, which should be considered for further exploration as a promising anti-cancer therapy.

**Supplementary Information:**

The online version contains supplementary material available at 10.1007/s42995-022-00162-x.

## Introduction

**Fig. 1:**
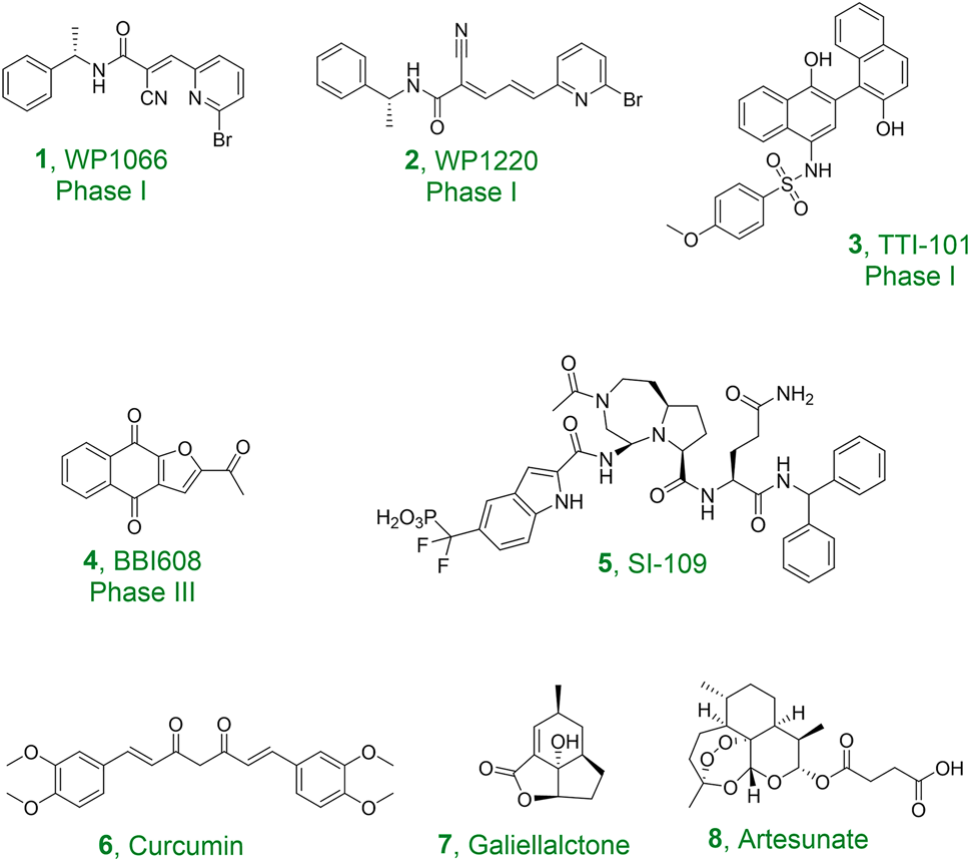
Chemical structures of representative STAT3 inhibitors

Signal transducer and activator of transcription 3 (STAT3) is a latent oncogenic transcription factor and belongs to the STAT protein family. It is comprised of six functional domains: N-terminal domain (NTD) crucial for oligomerization, coiled-coil domain (CCD) responsible for protein–protein interaction, DNA-binding domain (NBD) facilitating gene transcription, linker domain, Src-homology 2 domain (SH2) for STAT3 dimerization, and C-terminal transactivation domain (CTD) to recruit co-factors (Becker et al. 1998). STAT3 plays important roles in multiple cellular processes including proliferation, survival, apoptosis, and the transcription of target genes upon stimulation (Dong et al. 2021). Aberrant STAT3 activation has been observed in a wide range of cancers such as lung cancer (Zhang et al. 2019), colorectal cancer (Heichler et al. 2020), breast cancer (Siersbaek et al. 2020), melanoma (Swoboda et al. 2021), ovarian cancer (Geethadevi et al. 2021), prostate cancer (Culig 2017), hepatocellular carcinoma (He and Karin 2011), pancreas cancer (Fukuda et al. 2011), leukemia (Arora et al. 2018) and multiple myeloma (Zheng et al. 2021), and plays a central role in immune regulation (Zou et al. 2020). STAT3 has a long history of being studied as a target for anti-cancer therapeutics (Huang et al. 2020). Currently, there are several direct STAT3 inhibitors (e.g., compounds **1**–**4**, Fig. 1) in various stages of clinical trials (Ott et al. 2020). More small molecules are in the discovery stage, including synthetic compounds (e.g., compound **5**, Fig. 1) and natural products (e.g., compounds **6**–**8**, Fig. 1) which target the SH2 or DNA binding domain of STAT3 (Bai et al. 2019, Huang et al. 2021 and Zhong et al. 2022). However, no STAT3 inhibitor has been approved for clinical use although they are able to suppress tumor growth and induce apoptosis in laboratory studies. Thus, there is an urgent need for STAT3 targeting therapeutics that are more diverse in their pharmaceutical properties and chemical structures.

**Fig. 2:**
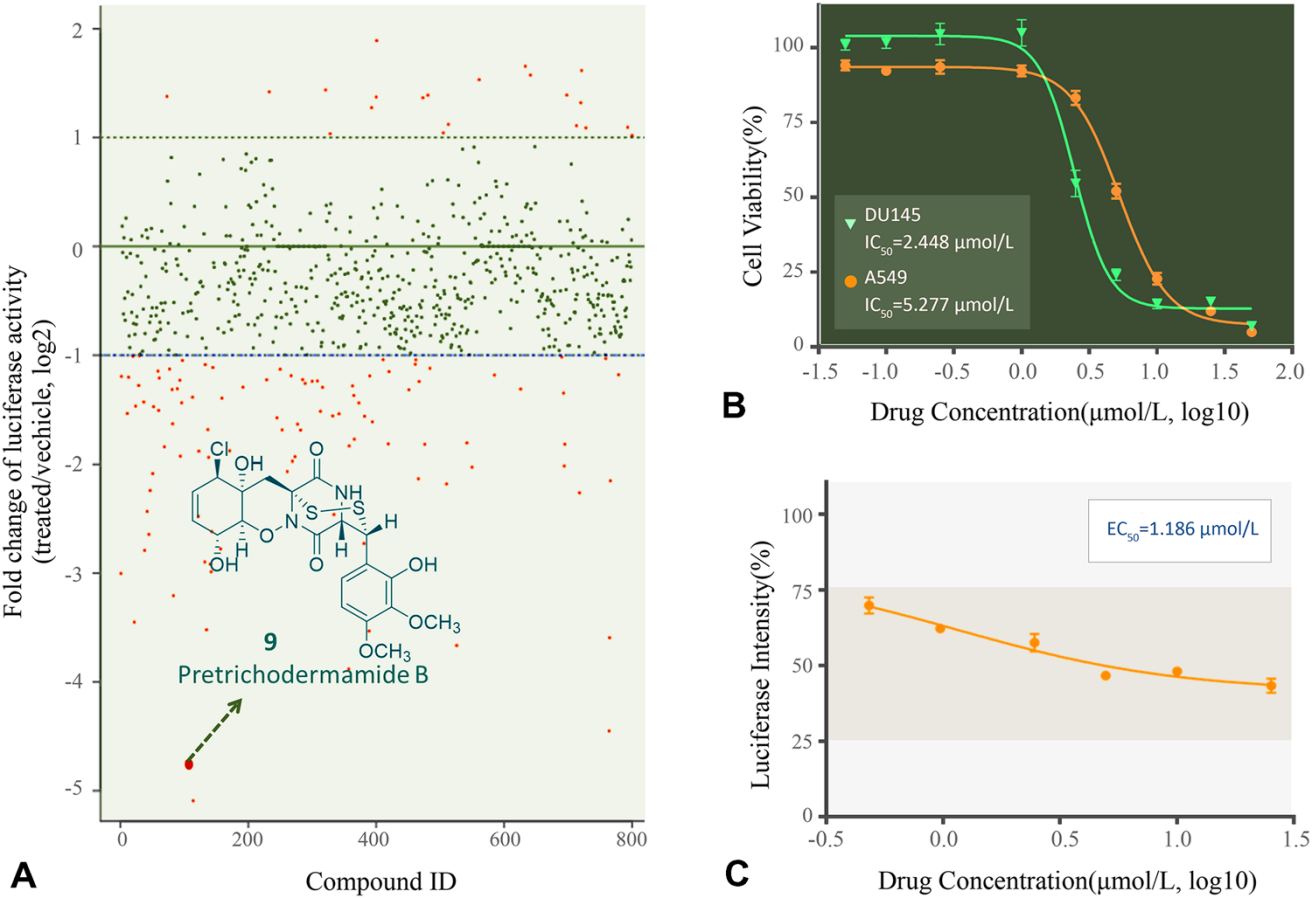
Pretrichodermamide B was identified as a JAK/STAT3 signaling inhibitor by a STAT3-based luciferase drug screening system. **A** Fold changes of screened marine natural products in a constitutive STAT3 activation-based luciferase assay. **B** Pretrichodermamide B suppressed the growth of cancer cells DU145 and A549, measured by MTT assay. **C** Pretrichodermamide B inhibited the luciferase intensity in a dose-dependent manner

Marine natural products (MNPs) have been considered as great resources for therapeutic discovery of lead compounds for cancer therapy and development of novel drug candidates (Hai et al. 2021; Xu et al. 2021; Nie et al. 2020). Our group has long been working on the isolation, structural elucidation, structure–activity-relationship (SAR) analysis, biological evaluation and mechanism interpretation of MNPs (Liu et al. 2019; Peng et al. 2021). In order to identify novel STAT3 inhibitors, we screened our in-house MNP library via a Janus kinase (JAK)/STAT3 signaling reporter assay we previously developed (Zhang et al. 2020). Pretrichodermamide B (compound **9**, Fig. 2A) stood out, which was an epipolythiodioxopiperazine containing a disulfide bridge and a unique *O*-alkyl-oxime fragment. It was originally discovered from *Trichoderma longibrachiatum* (Nakano et al. 1990) and re-isolated in our laboratory from a deep-sea derived fungus *Penicillium janthinellum*. Several studies were performed on its preliminary anti-cancer and anti-bacterial activities (Tang et al. 2020; Yamazaki et al. 2015), but none of them explored the detailed mode of action (MOA). Herein, we evaluated the in vitro anti-cancer activities of Pretrichodermamide B, identified its target proteins, predicted their binding modes, explored its effects on cell cycle as well as apoptosis, and confirmed its in vivo anti-cancer efficacy.

## Results and discussion

### Isolation and characterization of Pretrichodermanide B

*Penicillium janthinellum* SH0301 was cultured on rice solid medium at 25 °C for 60 days. The epipolythiodioxopiperazine Pretrichodermamide B was obtained via organic extraction and high performance liquid chromatography (Supplementary Fig. S1–S3). It was fully characterized by ^1^H NMR, ^13^C NMR and ESI–MS which were consistent with previous reports (Nakano et al. 1990).

### Pretrichodermamide B inhibits JAK/STAT3 pathway and has antiproliferative activity in vitro

Pretrichodermamide B displayed the most potent inhibitory activity among all the tested MNPs in the constitutively active STAT3 cell line SKA, derived from A549 (Zhang et al. 2020). Luciferase intensity was inhibited by Pretrichodermamide B in a dose-dependent manner with an IC_50_ value of 1.19 μmol/L **(**Fig. 2C**)**. To confirm its cellular efficacy induced by JAK/STAT3 signaling inhibition, STAT3 constitutively activated cancer cell lines DU145 and A549 were treated with Pretrichodermamide B for 72 h. The cell growth was suppressed by Pretrichodermamide B in a dose-dependent manner with IC_50_ values of 2.45 μmol/L and 5.28 μmol/L against DU145 and A549, respectively (Fig. 2B). These results suggest that Pretrichodermamide B can efficiently suppress the JAK/STAT3 signaling and is worth further study as an anti-tumor agent.

### STAT3 as the target protein of Pretrichodermamide B

**Fig. 3:**
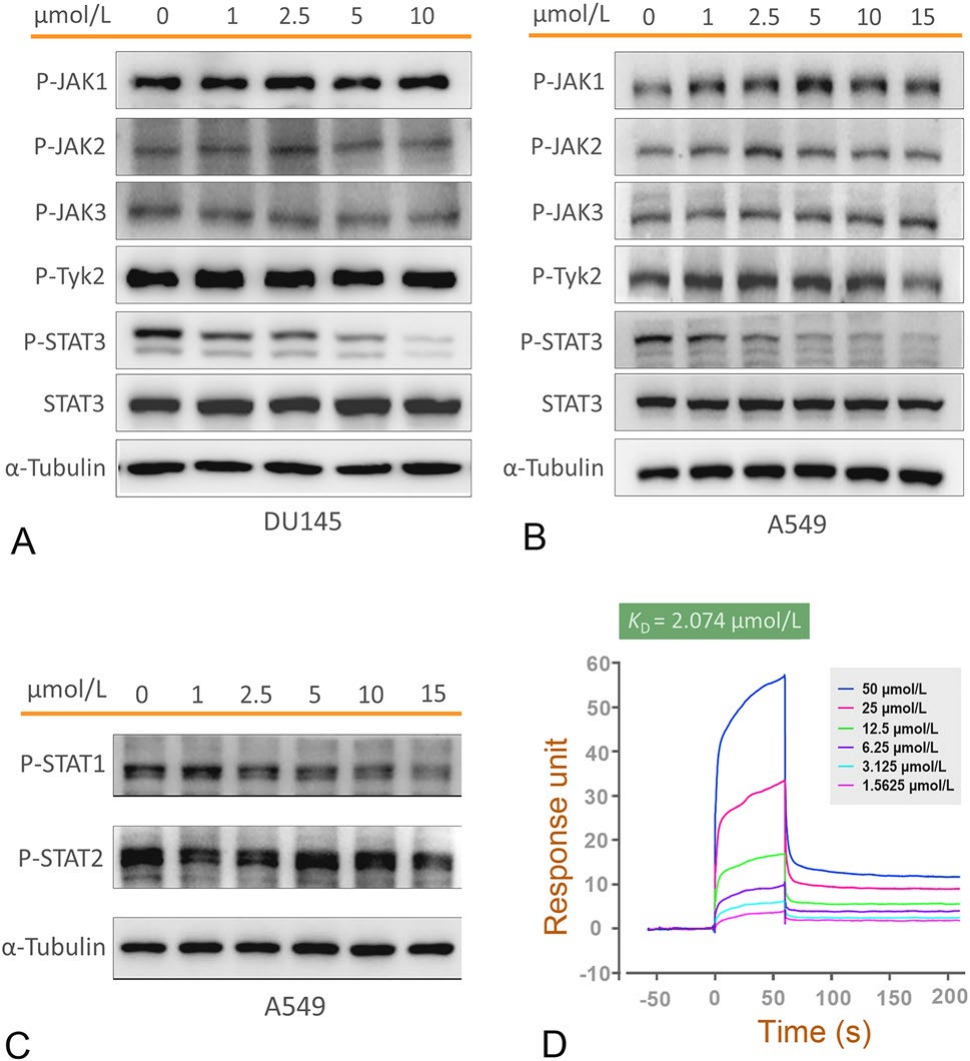
Pretrichodermamide B directly binds to STAT3 and blocks JAK/STAT3 signaling pathway via suppressing STAT3 phosphorylation. **A** DU145 cells were treated with Pretrichodermamide B at the indicated concentrations for 2 h, and the levels of phosphorylated JAK kinases (JAK1, JAK2, JAK3 and Tyk2), phosphorylated STAT3 and unphosphorylated STAT3 were examined by Western blot. **B** A549 cells were treated with Pretrichodermamide B at the indicated concentrations for 2 h, and the levels of phosphorylated JAK kinases, phosphorylated STAT3 and unphosphorylated STAT3 were demonstrated by Western blot. **C** A549 cells were administrated with Pretrichodermamide B at the indicated concentrations for 2 h, and the levels of phosphorylated STAT1 and STAT2 were determined by Western blot. **D** Binding affinity of Pretrichodermamide B with STAT3 was determined by SPR

In order to elucidate the direct target proteins, we examined the effects of Pretrichodermamide B on phosphorylation of multiple proteins involved in the JAK/STAT3 signaling pathway by Western blot. As shown in Fig. 3, Pretrichodermamide B had little influence on the phosphorylation of JAK family proteins (e.g., JAK1 ~ 3 and tyrosine kinase 2 (Tyk2)) in DU145 cells; however, it significantly inhibited the STAT3 phosphorylation (Y705) in a dose dependent manner without obvious effect on total protein level of STAT3 at 1 μmol/L (Fig. 3A), which indicates that Pretrichodermamide B might influence STAT3 phosphorylation independent of its upstream JAK kinases activation. Similar results were observed in the A549 cell line (Fig. 3B). Further exploration showed that Pretrichodermamide B can also slightly decrease the phosphorylation of STAT1 at 10 μmol/L, while no obvious effect on STAT2 phosphorylation was observed at the high dose of 15 μmol/L (Fig. 3C), suggesting that Pretrichodermamide B mediates the downregulation of STAT3 phosphorylation, while having little effect on other members of the STAT family. This observation further emphasizes the possibility that Pretrichodermamide B might interact directly with STAT3 itself. Thus, the direct binding of Pretrichodermamide B with STAT3 was investigated by surface plasmon resonance (SPR) spectroscopy. The results supported our hypothesis that Pretrichodermamide B directly binds with STAT3 protein, and the binding constant was 2.07 μmol/L (Fig. 3D).

### Molecular docking of Pretrichodermamide B with STAT3

**Fig. 4:**
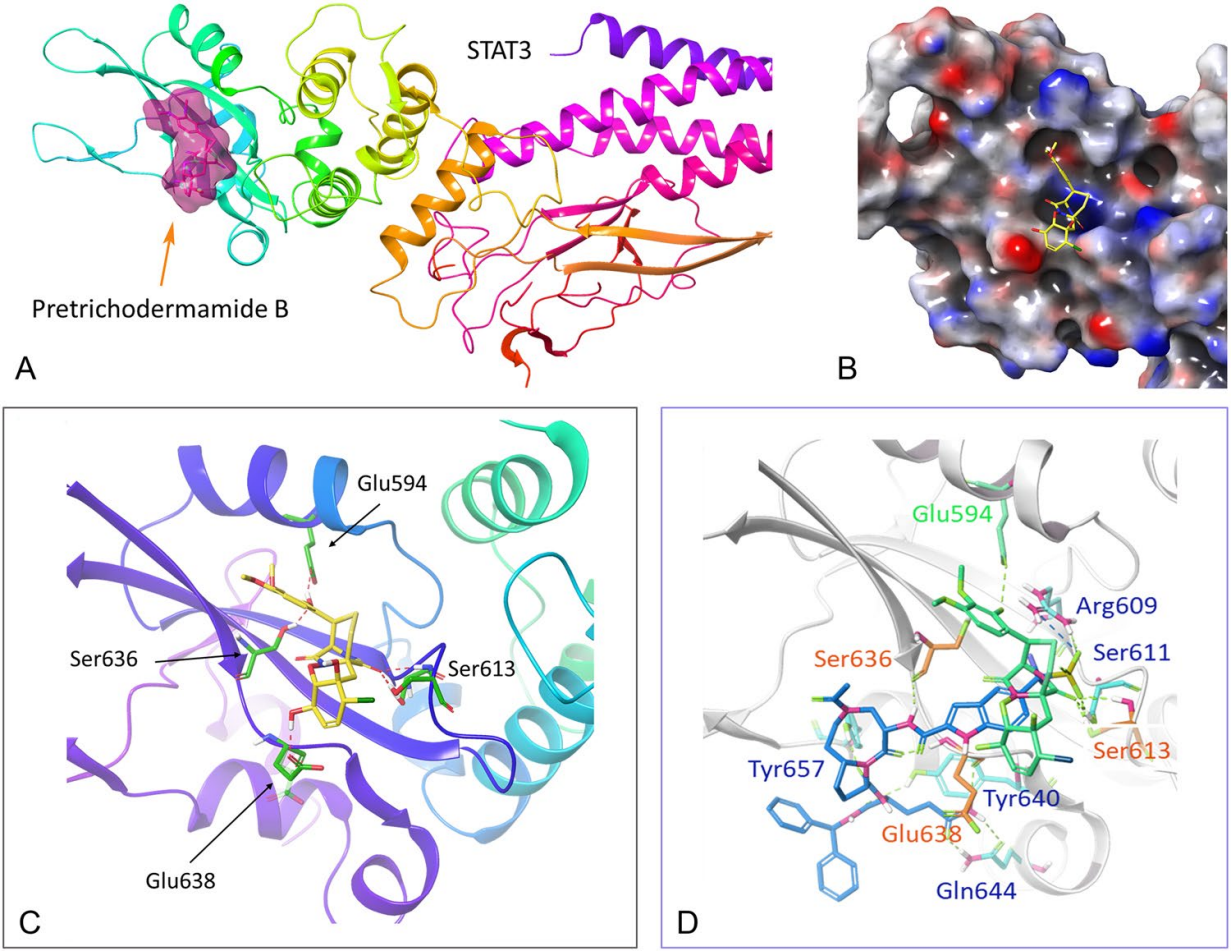
**A** Ribbon representation of Pretrichodermamide B (highlighted in magenta) docked into STAT3 (PDB code: 6NJS). **B** Electrostatic potential surface representation of the docking results. **C** Hydrogen bonds (dashed lines colored in red) interactions between STAT3 and Pretrichodermamide B. Involved residues Glu594, Ser613, Ser636 and Glu638 are colored in green. **D** Superimposition of Pretrichodermamide B (green) and compound **5** (blue) with STAT3. The residues of STAT3 interacting with Pretrichodermamide B are highlighted in green, and residues interacting with compound **5** are colored in blue while their shared interacting residues are colored in orange

Molecular docking studies of Pretrichodermamide B with STAT3 (PDB code: 6NJS) were conducted using Schrödinger Small-Molecule Drug Discovery Suite (Fig. 4). Pretrichodermamide B occupies the SH2 binding pocket of STAT3 very well with two extended deep pockets (Fig. 4A,B). The OH group on the aromatic ring of Pretrichodermamide B forms two direct hydrogen bonds with Glu594 and Ser636. The other OH group on the aliphatic ring interacts with Glu638 directly via a hydrogen bond, while the oxygen on diketopiperazine forms three hydrogen bonds with Ser611 and Ser613 (Fig. 4C). Compared to compound **5** (SI109, PDB code: 6NUQ), the critical interactions with Ser636, Glu638 and Ser613 are similar while compound **5** does not extend to the pocket Glu594 is located (Fig. 4D). At the same time, compound **5** interacts with Tyr657, Tyr640 and Glu638 due to the large size which provides great insight on how to modify Pretrichodermamide B in the near future. In a word, these docking results indicated that Pretrichodermamide B may disrupt the formation of STAT3 dimer through occupying the STAT3 SH2 domain.

### Pretrichodermamide B induces cell cycle arrest and promotes cell apoptosis

**Fig. 5:**
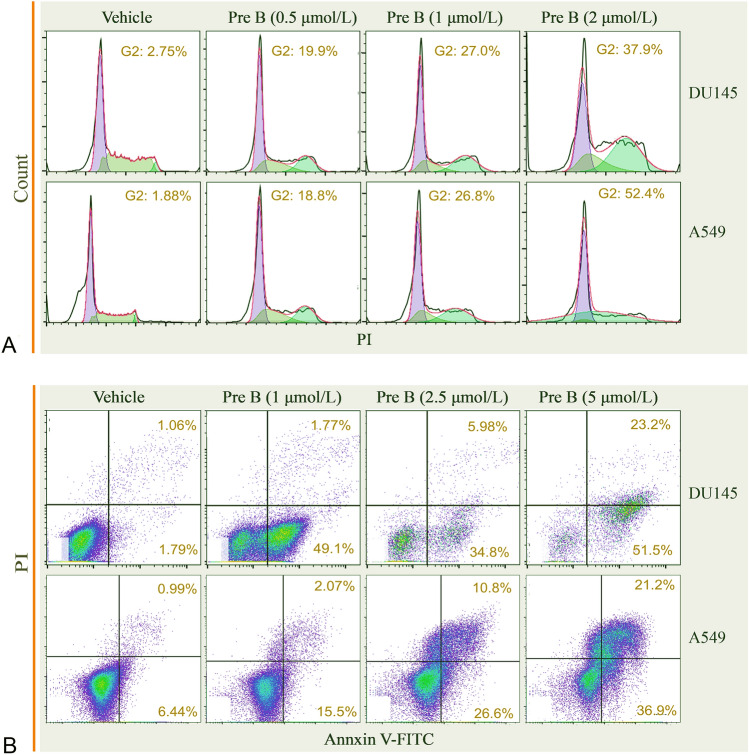
Pretrichodermamide B (Pre B) caused G2 cell cycle arrest and induced apoptosis. **A** DU145 and A549 cells were treated with vehicle and Pretrichodermamide B at the indicated concentrations for 24 h and then processed for cell cycle analysis. **B** DU145 and A549 cells were treated with vehicle or Pretrichodermamide B at the indicated concentrations for 48 h for apoptosis analysis by Annexin V-FITC/PI assay

Considering the important roles of JAK/STAT3 signaling in cell proliferation and survival, which were achieved through modulating the transcription of cell cycle-relevant genes (e.g., *cyclin D* and *cyclin B*) as well as antiapoptotic genes (e.g., *Bcl-x* and *Bcl-2*) (Fukada et al. 1998; Hirano et al. 2000), we analyzed the effects of Pretrichodermamide B on cell cycle progression in DU145 and A549 cells. Illustrated in Fig. 5A, Pretrichodermamide B at the dose of 1 μmol/L was able to cause DU145 cell cycle arrest at the G2 phase (vehicle, 2.75% in G2 phase; 1 μmol/L Pretrichodermamide B, 27.0%; and 2 μmol/L Pretrichodermamide B, 37.9%). The effects on A549 cell cycle arrest were similar (vehicle, 1.88% in G2 phase; 1 μmol/L Pretrichodermamide B, 26.8%; and 2 μmol/L Pretrichodermamide B, 52.4%). Moreover, Pretrichodermamide B induced apoptosis in DU145 and A549 cells in a dose-dependent manner. The percentage of DU145 cells in early apoptosis (Annexin V+/PI−) in the vehicle group was 1.79%; whereas, in the 0.5, 1 and 2 μmol/L of Pretrichodermamide B treated groups, the apoptosis percentages were 49.1%, 34.8% and 51.5%, respectively. In addition, the rate of DU145 cells in late apoptosis (Annexin V+/PI+) was 1.06% for the vehicle treatment group and 1.77%, 5.98%, and 23.2% for the 0.5, 1 and 2 μmol/L, respectively, of Pretrichodermamide B treatment groups (Fig. 5B). The apoptosis inducing effects of Pretrichodermamide B in A549 cells were consistent with that in DU145 cells. This data suggested that Pretrichodermanide B could influence STAT3 activation as well as regulate downstream cell functions.

### In vivo evaluation of antitumor activity of Pretrichodermamide B in a xenograft mouse model

As STAT3 is overactivated in the majority of solid tumors, we evaluated the potential in vivo therapeutic efficacy of Pretrichodermamide B in an A549 cell line derived xenograft (CDX) mouse model. As shown in Supplementary Fig. S4, 10 mg/kg of Pretrichodermamide B via intraperitoneal (i.p.) administration significantly inhibited A549 tumor growth compared with the vehicle treatment. Obvious tumor growth inhibition (~ 40%) of Pretrichodermamide B at the dose of 10 mg/kg was observed, which was comparable to the efficacy of Gefitinib at the dose of 50 mg/kg by oral administration. Meanwhile, there is no notable changes of body weight or observable liver and kidney injuries in Pretrichodermamide B treated mice (Supplementary Fig. S4B and S4E), suggesting that administration of Pretrichodermamide B at the indicated dose had no or relatively low toxicity.

## Conclusions

In summary, we have identified a small molecular MNP, Pretrichodermamide B, as a potent JAK/STAT3 signaling inhibitor through a STAT3-based luciferase drug screening system. Pretrichodermamide B was able to suppress cell proliferation, promote cell apoptosis and cause cell cycle arrest at the G2 phase in two STAT3 constitutively activated cell lines (A549 and DU145) at low micromolar concentrations. SPR identified STAT3 as the direct binding target of Pretrichodermamide B, and binding models with critical interactions were revealed by a computational docking study. Significant tumor growth reduction, in vivo*,* using an A549-CDX model was observed with no obvious signs of toxicity. This suggests that Pretrichodermamide B has great therapeutic potential as an anti-tumor agent. More studies on physiochemical parameters as well as the pharmacokinetic properties of Pretrichodermamide B are ongoing.

## Materials and methods

### Materials

Antibodies against p-Tyr701-STAT1 (product #9167), p-Tyr690-STAT2 (product #88410), p-Tyr705-STAT3 (product #9145), p-Tyr1022/1023-JAK1 (product #3331), p-Tyr1007/1008-JAK2 (product #3776), and p-Tyr1054/1055-Tyk2 (product #9321) were purchased from Cell Signaling Technology, USA. Recombinant human STAT3 was obtained from Abcam, UK (product #ab268982). Protease inhibitors and phosphatase inhibitors were obtained from Merck Life Science, USA. Marine natural products library was built in house in the Laboratory for Marine Resources Biology at Ocean University of China.

Six-week old female NRMI congenitally athymic nude (nu/nu) mice (SPF degree, 17–20 g weight) were purchased from Beijing Vital River Laboratory Animal Technology Co., Ltd.

### Isolation of Pretrichodermamide B

The fungal strain was cultured on rice solid medium (Jinlongyu, China) following the previous reported procedure (Yang et al. 2020). Then the crude organic extract was divided into five fractions (Fr.1–Fr.5) via silica gel vacuum liquid chromatography (VLC). Fr.3 was separated by an octadecyl silane column (K08670357, Sweden, product #K08670357) and then purified by recrystallization to obtain Pretrichodermamide B (548 mg). ^1^H NMR and ^13^C NMR of Pretrichodermamide B are consistent with previously reported data (Nakano et al. 1990). ^1^H NMR (400 MHz, DMSO-*d*_6_) *δ* 9.45 (*s*, 1H), 9.12 (*d*, *J* = 4.6 Hz, 1H), 7.43 (*d*, *J* = 8.8 Hz, 1H), 6.55 (*d*, *J* = 8.8 Hz, 1H), 5.71–5.53 (*m*, 3H), 5.40 (*s*, 1H), 4.87 (*d*, *J* = 2.5 Hz, 1H), 4.51 (*d*, *J* = 2.8 Hz, 1H), 4.44 (*t*, *J* = 3.8 Hz, 1H), 4.39–4.29 (*m*, 1H), 4.06 (*s*, 1H), 3.78 (*s*, 3H), 3.67 (*s*, 3H), 2.18 (*d*, *J* = 15.8 Hz, 1H), 2.06 (*d*, *J* = 15.6 Hz, 1H). Purity of Pretrichodermamide B (> 95%) was established by analytical HPLC, which was carried out on a Waters alliance HPLC system.

### Cell culture

SKA cells which were derived from A549 cells constructed with a STAT3-driven luciferase reporter gene were cultured in DMEM medium (Zhang et al. 2019); whereas, DU145 and A549 cells were cultured in RPMI 1640 medium. Both media were supplemented with 10% fetal bovine serum, streptomycin (100 mg/mL) and penicillin (100 IU/mL) (Procell, China). All the cells were maintained at 37 °C in humidified incubators containing 5% CO_2_.

### STAT3-dependent reporter assay

SKA cells (10^4^ cells/well) were seeded into white 96-well plates and maintained overnight at 37 °C in an incubator containing 5% CO_2_. Then the cells were treated with the listed compounds at the indicated concentrations for 24 h. Luciferase activity was determined using luciferase detection kits (Promega, USA, product #E1483) and detected by a SpectraMax®L microplate reader (Molecular Devices, USA).

### Cell viability assay

Cells at the density of 4000/well were seeded into 96-well plates. After 18 h, the cells were treated with either vehicle or the listed compounds at the indicated concentrations. Seventy-two hrs later, 10 μL of resazurin (1 mg/mL, Abcam, USA, product #ab129732) was added to each well and incubated for another 3 h. The absorbance was measured at 595 nm emission wavelength and 544 nm excitation wavelength by a SpectraMax@i3 enzyme-labeled instrument (Molecular Devices, USA).

### Western blotting

The cultured cells were lysed by RIPA buffer. Protein lysates were separated via SDS-PAGE and transferred onto nitrocellulose membranes (GE Healthcare, USA). The membranes were probed by primary antibodies and then incubated with horseradish peroxidase-conjugated secondary antibodies (Sungene, China, LK2001). Immune complexes were detected by an Immobilon™ western chemiluminescence HRP substrate (Millipore, USA, product #WBKLS0500) and photographed with a Tanon 5200 imaging system.

### SPR assay

Surface plasmon resonance (SPR) analysis was conducted with a Biacore T200 instrument (GE Healthcare, USA) with CM5 sensor chip (GE Healthcare, USA). To test the binding affinity between Pretrichodermamide B and STAT3 127–722 protein, serially diluted concentrations of Pretrichodermamide B were injected into the fluid flow system. Phosphate buffered saline (PBS) and the analyte was injected at the fluid flow rate of 30 μL/min. The experiment was performed at 25 °C with 60 s of association time and 150 s of dissociation time. Since Pretrichodermamide B was dissolved in 5%DMSO-95%PBS solution, a solvent correction assay was performed to adjust the results. STAT3 protein was immobilized on the sensor chip (CM5) using the amine-coupling method following standard procedures. Pretrichodermamide B at various concentrations was injected into the fluid flow system. The *K*_D_ values were calculated by Biacore T200 plus evaluation software using the kinetics and affinity analysis option (version 3.0).

### Flow cytometry analysis

DU145 or A549 cells at the density of 5 × 10^5^/well were seeded into 6-well plates and treated with vehicle, serum-free medium, or Pretrichodermamide B at the indicated concentrations. The cells were then harvested and stained with a Cell Cycle Staining Kit (Lianke Bio, China, product #CCS012) after 24 h of treatment and an eBioscience™ Annexin V-FITC Apoptosis Kit (Invitrogen, USA, product #BMS500FI-100) after 48 h of treatment, respectively. The states of the cells were observed by BD FACSAria III flow cytometry.

### Tumor xenograft model and in vivo antitumor assay

Six-Week-old male nude mice were randomized into one of five treatment groups (*n* = 7/group). Once the tumors were palpable, vehicle, Gefitinib (50 mg/kg, p.o., qod), or Pretrichodermamide B (2.5, 5 or 10 mg/kg, i.p., qod) were administrated. Tumor volume and body weight were monitored (tumor volume = 1/2 length × width^2^). After the treatments, mice were killed, and tumors were isolated, photographed, and weighted. The liver and kidney samples were fixed with formaldehyde and sectioned for H&E staining.

### Statistical analyses

Prism version 6 was utilized to perform the statistical analyses. One-way ANOVA was used to calculate the *p*-values in Supplementary Fig. S4C. Statistical significance was established for *p* < 0.033 (*), *p* < 0.002 (**) and *p* < 0.001 (***). The data are represented graphically as the mean ± SEM.

### Computational docking studies

The computational docking study was performed by Maestro of Schrödinger software. The crystal structure of STAT3 (PDB code: 6NJS) was obtained from PDB Bank and prepared with Protein Prepared Wizard. The polar hydrogens were added, crystal waters far away from the binding site were removed, and partial charges were assigned. The 3D structures of Pretrichodermamide B were created by Chemdraw, and the initial lowest energy conformations were calculated with Lig-Prep of Schrödinger Maestro. For all dockings, a 20 × 20 × 20 Å grid box with the grid center chosen on the centroid of native ligand of PDB structure and XP protocol were used. The Docking results were visualized via Schrödinger Maestro to demonstrate the ligand-receptor interactions.

## Supplementary Information

Below is the link to the electronic supplementary material.

Supplementary file1 (DOC 6589 KB)

## Data Availability

The data that supports the findings of this study are included in this published article (and its supplementary information file).
