# Supplementary material for: Identification of marine natural product Pretrichodermamide B as a STAT3 inhibitor for efficient anticancer therapy
Source: Mar Life Sci Technol. 2023 Feb 6;5(1):94–101. doi: 10.1007/s42995-022-00162-x (PMC10077262; doi:10.1007/s42995-022-00162-x)
Supplement: Supplementary file 1 — Supplementary file1 (DOC 6589 KB) [file 42995_2022_162_MOESM1_ESM.doc]

**Identification of marine natural product Pretrichodermamide B as a STAT3 inhibitor for efficient anticancer therapy**

Rui Li,^a,b,1^ Yue Zhou,^a,b,1^ Xinxin Zhang,^a,b^ Lujia Yang,^a,b^ Jieyu Liu,^a,b^ Samantha M. Wightman,^c^ Ling Lv,^a,b^ Zhiqing Liu,^a,b^,* Chang-Yun Wang^a,b^,* and Chenyang Zhao^a,b^,*

^a^School of Medicine and Pharmacy, Institute of Evolution & Marine Biodiversity, College of Food Science and Engineering, Ocean University of China, Qingdao 266003, China.

^b^Laboratory for Marine Drugs and Bioproducts, Qingdao National Laboratory for Marine Science and Technology, Qingdao 266237, China

^c^Department of Cancer Biology, Lerner Research Institute, Cleveland Clinic, Cleveland, OH 44195, USA

^1^These authors contribute equally to this work.

**Corresponding author:**

*Chenyang Zhao, PhD

Email: [zhaochenyang2021@gmail.com](mailto:zhaochenyang2021@gmail.com)

*Chang-Yun Wang, PhD

Email: [changyun@ouc.edu.cn](mailto:changyun@ouc.edu.cn)

*Zhiqing Liu, PhD

Email: [liuzhiqing@ouc.edu.cn](mailto:liuzhiqing@ouc.edu.cn%0D)

**Contents**

| Fig. S1 ^1^H NMR spectra of Pretrichodermamide B………………….... | 3 |
| --- | --- |
| Fig. S2 ^13^C NMR spectra of Pretrichodermamide B………................... | 3 |
| Fig. S3 HPLC profile of Pretrichodermamide B………………………. | 4 |
| Fig.S4 *In vivo* efficacy of Pretrichodermamide B on A549 cell line derived xenograft mouse model………………………………………... | 5 |
|  |  |

Pretrichodermamide B, white solid. ^1^H NMR (400 MHz, DMSO-*d*_6_) *δ* 9.45 (s, 1H), 9.12 (d, *J* = 4.6 Hz, 1H), 7.43 (d, *J* = 8.8 Hz, 1H), 6.55 (d, *J* = 8.8 Hz, 1H), 5.71 – 5.53 (m, 3H), 5.40 (s, 1H), 4.87 (d, *J* = 2.5 Hz, 1H), 4.51 (d, *J* = 2.8 Hz, 1H), 4.44 (t, *J* = 3.8 Hz, 1H), 4.39 – 4.29 (m, 1H), 4.05 (d, *J* = 7.4 Hz, 1H), 3.78 (s, 3H), 3.67 (s, 3H), 2.18 (d, *J* = 15.8 Hz, 1H), 2.06 (d, *J* = 15.6 Hz, 1H). ^13^C NMR (126 MHz, DMSO-*d*_6_) *δ* 167.05, 164.88, 153.39, 148.26, 136.24, 131.71, 126.96, 123.40, 116.73, 103.72, 85.93, 70.48, 67.60, 64.58, 60.71, 59.28, 56.12, 45.36, 33.54. ESIMS m/z 517.1 [M+H]^+^.

**Supplementary Fig. S1** ^1^H NMR of Pretrichodermamide B

**Supplementary Fig. S2** ^13^C NMR of Pretrichodermamide B


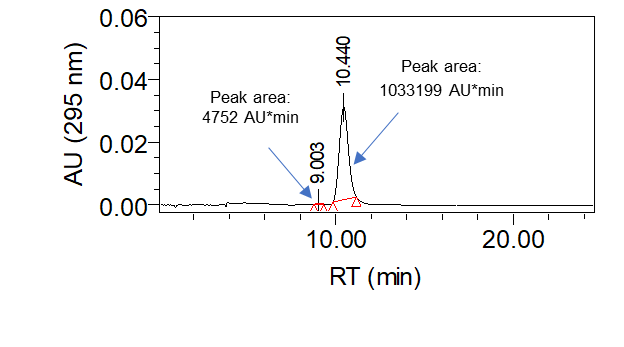


**Supplementary Fig. S3** HPLC profile of Pretrichodermamide B. Purity of Pretrichodermamide B (99.5%, *t*_R_ = 10.44 min, Figure S3) was established by analytical HPLC, which was carried out on a Waters Alliance HPLC System (model: Waters 1525, Waters 996 photodiode array detector). HPLC analysis conditions: Kromasil C18 (250 × 10 mm); flow rate 2 mL/min; UV detection at 295 nm; isocratic elution with 50% MeOH in water for 20 min. The purity is calculated as percentage of target peak area (*t*_R_ = 10.44 min) in relation to total area of peaks under baseline.


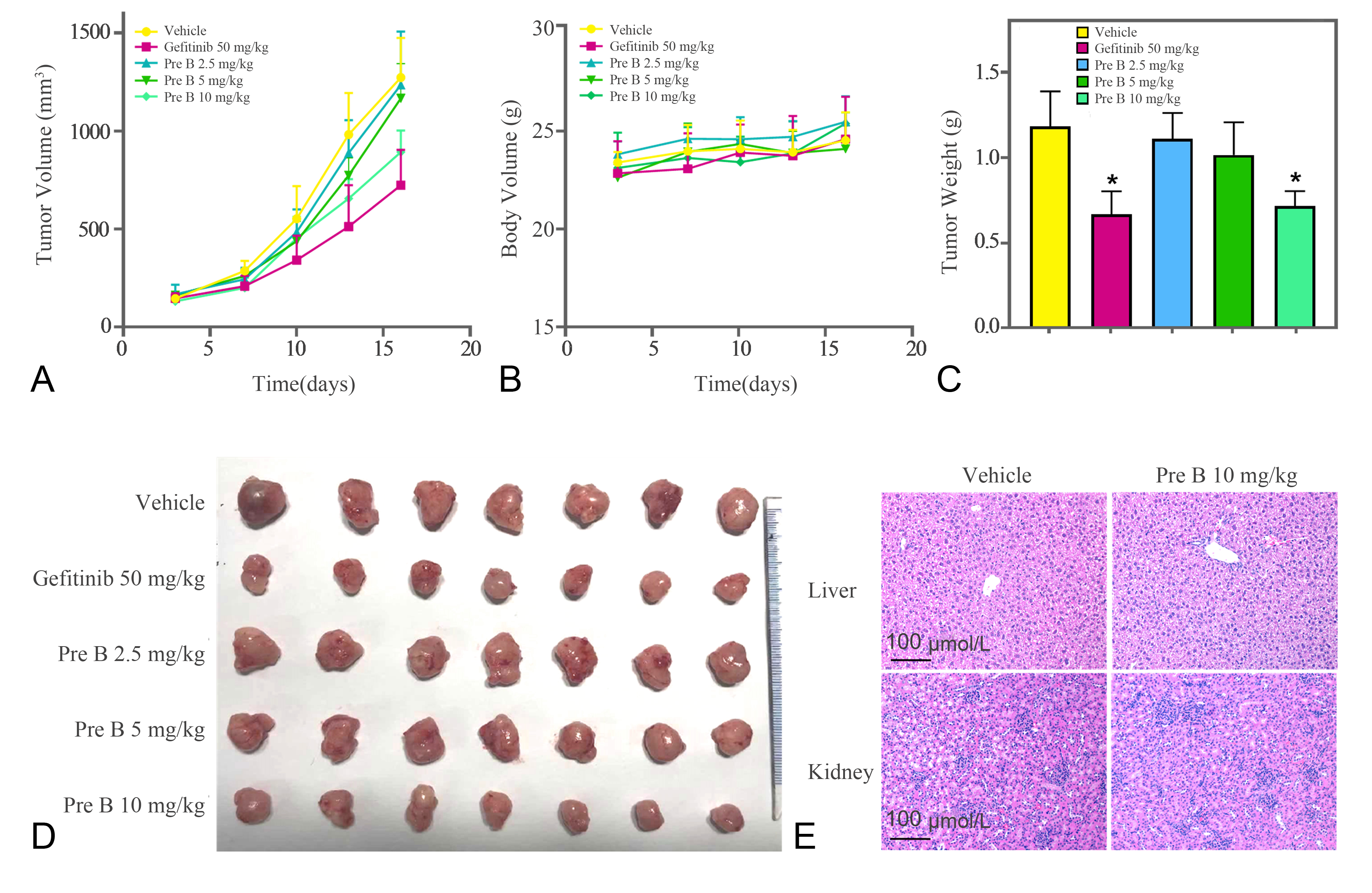


**Supplementary Fig. S4** *In vivo* efficacy of Pretrichodermamide B on A549 cell line derived xenograft mouse model. A549 xenograft (flank) nude mice were treated with vehicle, gefitinib (50 mg/kg, p.o.) every other day (qod) or Pretrichodermamide B (2.5, 5 or 10 mg/kg, i.p., qod) for 16 days. A) Monitored tumor volume of four groups. B) The tumor volumes were calculated before excision. One-way ANOVA was performed, p < 0.033 (*). C) Tumors were excised and photographed. D) The body weights during drug treatment are shown. E) H&E staining of the liver and kidneys of treated mice in vehicle and 10 mg/kg of Pretrichodermamide B treatment groups.
